# Supplementary figures and images for: ZNF423 patient variants, truncations, and in-frame deletions in mice define an allele-dependent range of midline brain abnormalities
Source: PLoS Genet. 2020 Sep 14;16(9):e1009017. doi: 10.1371/journal.pgen.1009017 (PMC7515201; doi:10.1371/journal.pgen.1009017)

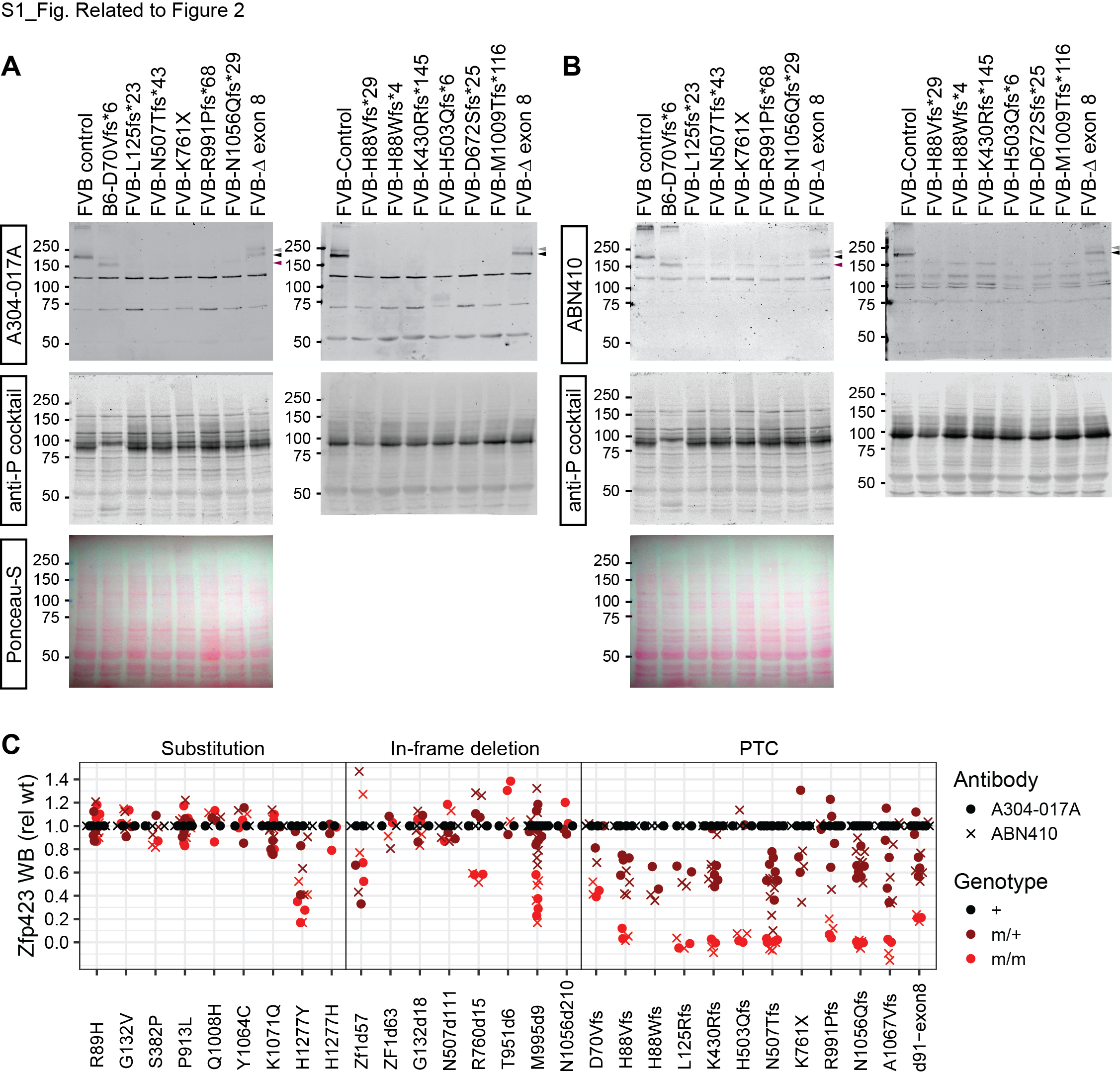

Supplement: S1 Fig — Western blot loading controls and quantification. (A) Full images for N-terminal blots in Fig 2A. A304-017A antibody detects Zfp423 (black arrowhead) and a variable conformational isomer (gray arrowhead) in control samples as well as a D70Vfs*6-specific protein (purple arrowhead). Molecular weight (kDa) of size marker bands is shown to the left. Membranes re-probed with a cocktail of antibodies againt phosphoserine, phosphothreonine, and phosphotyrosine show approximately even loading. The major band difference in D70Vfs sample was shown in other blots to be a difference between B6 and FVB strain backgrounds. Ponceau-S staining of the membrane before antibody application also shows approximately equal loading. (B) Western blots and stained membranes used for C-terminal antibody ABN410. (C) Approximate quantification by infrared imaging of all blots in this work. All measures adjusted to loading controls and plotted as ratio to wild-type control sample on the same membrane. Dots are measure from A304-017A, crosses from ABN410. Colors indicate genotypes with non-mutant controls in black, heterozygotes in brown and homozygous mutants in red. (TIF) [file pgen.1009017.s008.tif]

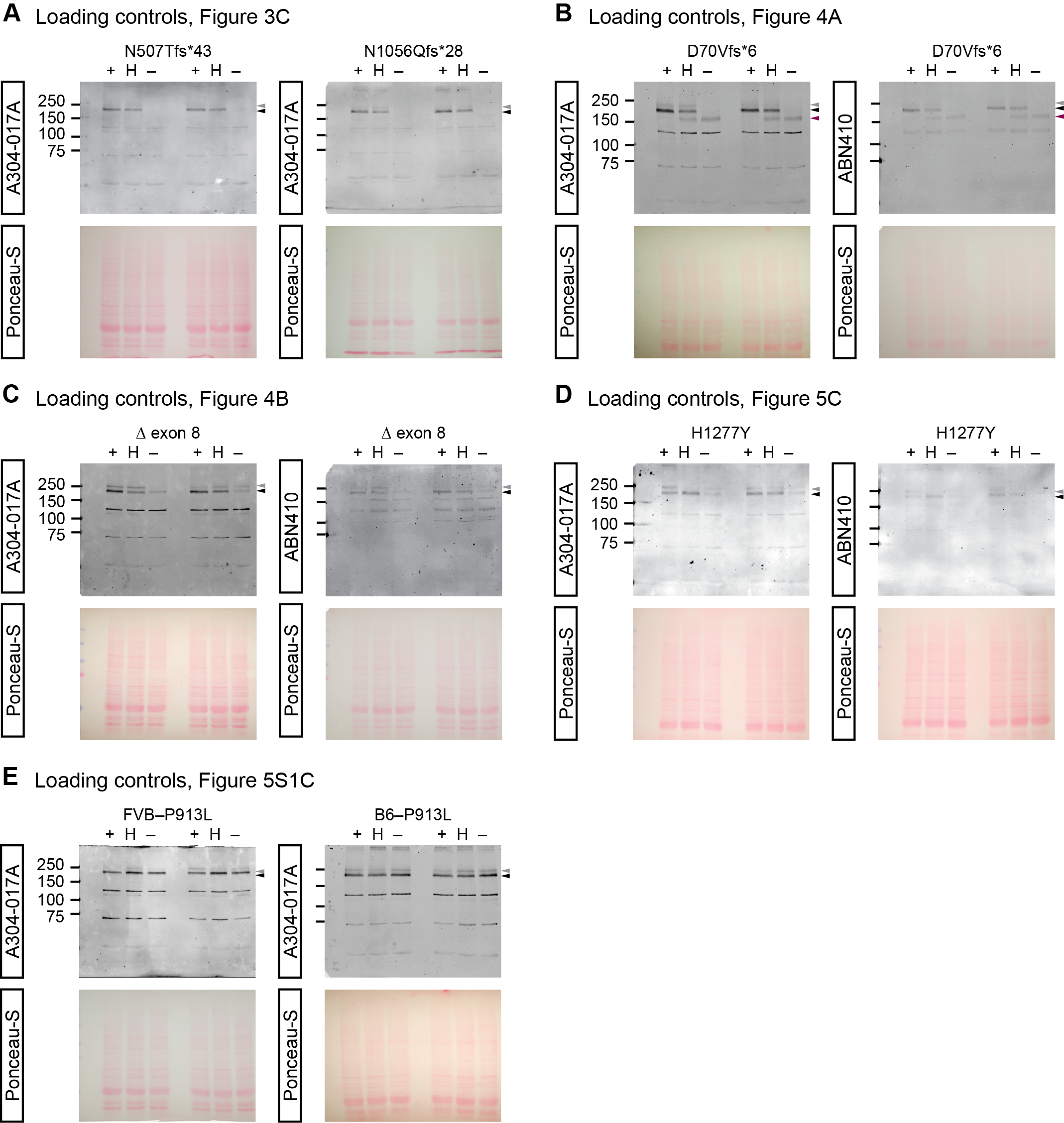

Supplement: S2 Fig — Western blots and loading controls. Full blots and Ponceau-S stained membranes for blots shown in Fig 3C (A), Fig 4A (B), Fig 4B (C), 5C (D) and S3C Fig. Size marker molecular weight in kDa is shown to the left. Position of the primary Zfp423 band is indicated by a black arrowhead to the right of the blot and the inconsistent conformational isomer by a gray and any consistently observed mutant specific band is indicated by a purple arrowhead. (TIF) [file pgen.1009017.s009.tif]

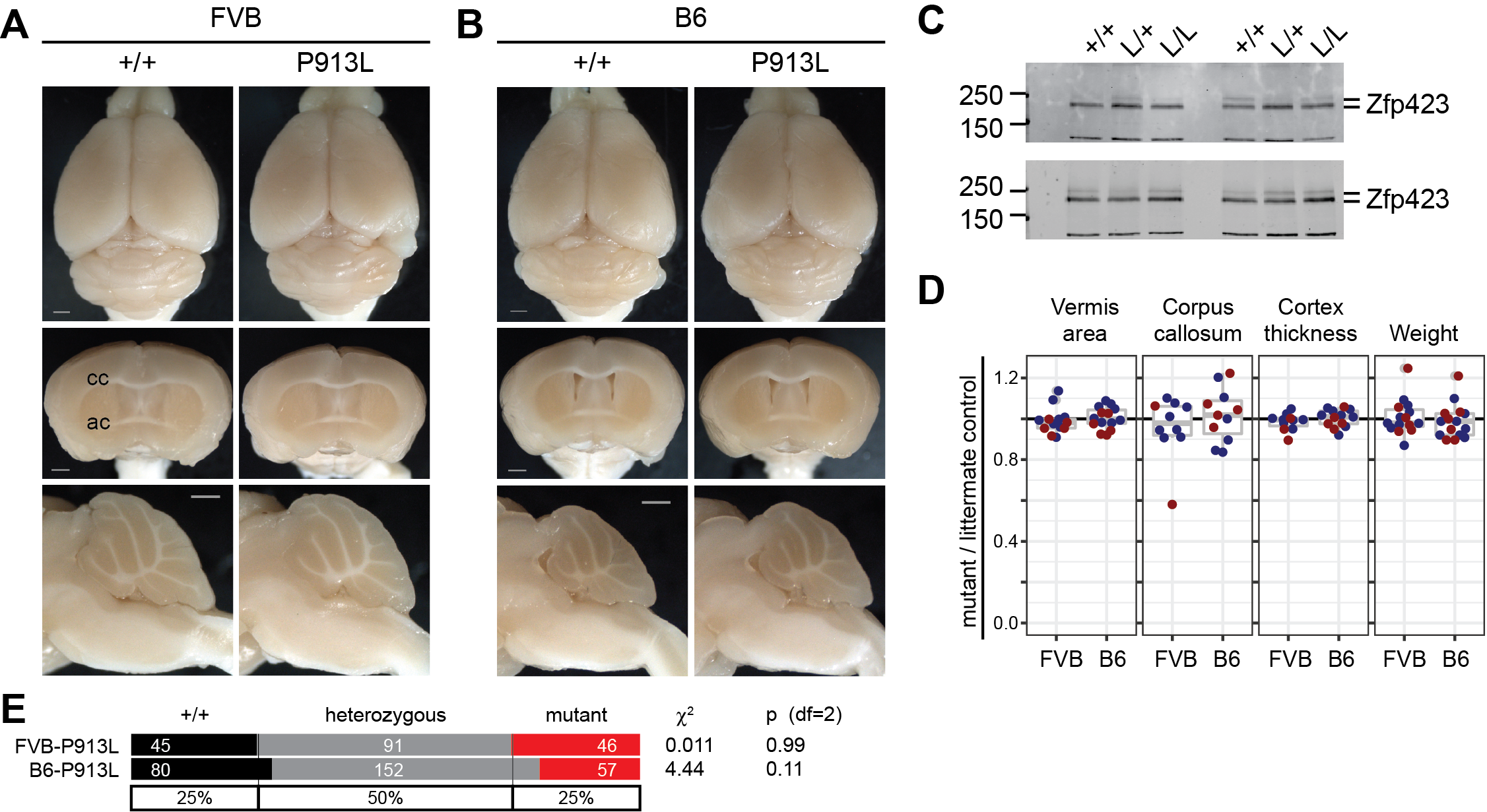

Supplement: S3 Fig — P913L is not pathogenic in mice. (A) Surface views of brains from control and mutant same-sex littermate pairs shows grossly normal brains for P913L substitution allele edited independently on FVB/NJ and C57BL/6 (B6) strain backgrounds. (B) Forebrain images show apparently normal structure for P913L mutant on both backgrounds, while highlighting different extent of lateral ventricles between strains at this place of section. (C) Western blots for FVB-P913L (top) or B6-P913L (bottom) with antibody against residues 250–300 (A304-017A) show normal Zfp423 protein abundance. Results from two distinct trios shown. Ratios between same-sex littermates for (D) vermis area at midline, corpus callosum thickness at midline, average cortical thickness at 15°, 30° and 45° from midline, and body weight fail to identify defects in P913L homozygotes. Scale bars, 1 mm. (TIF) [file pgen.1009017.s010.tif]

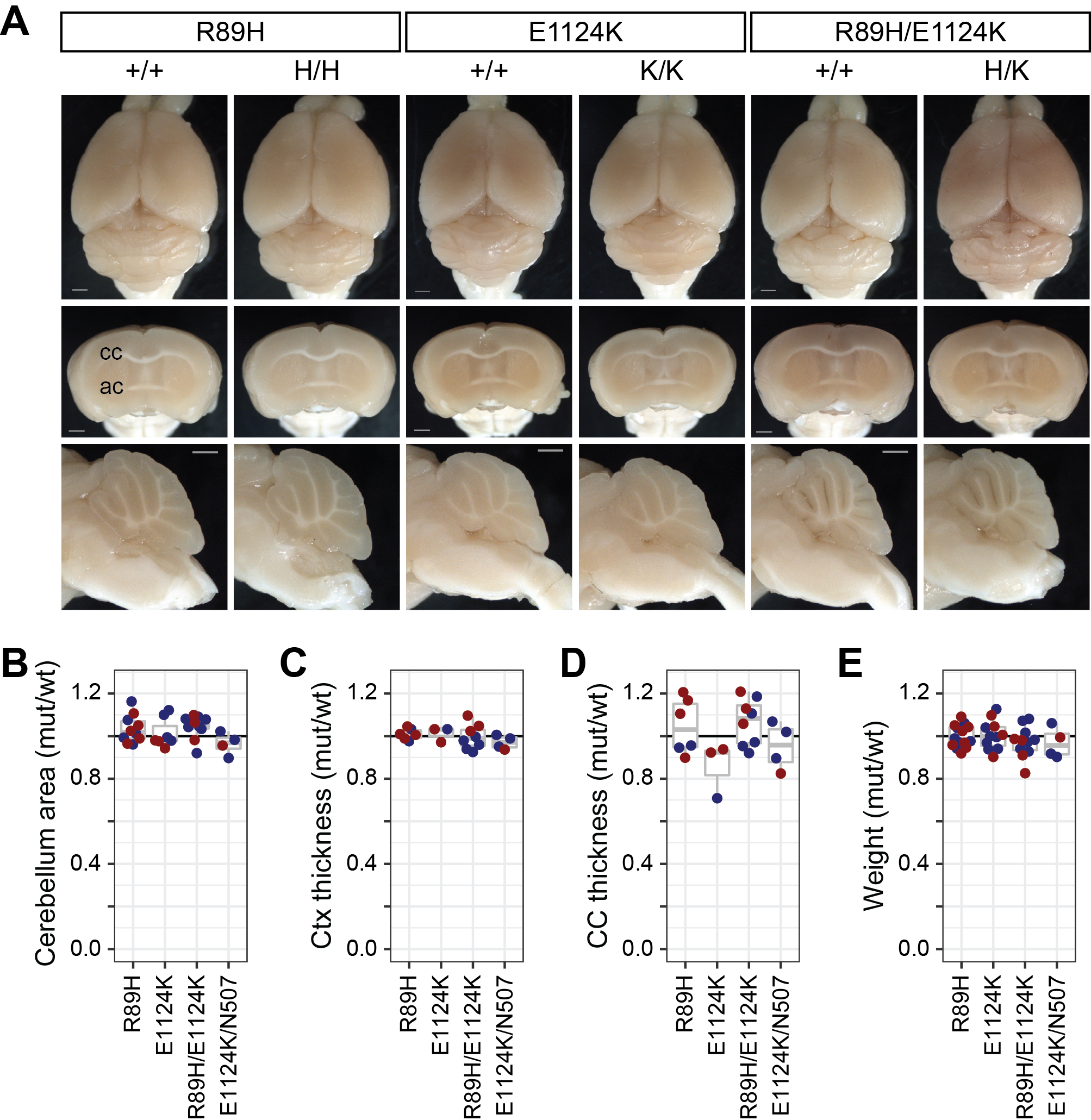

Supplement: S4 Fig — R89H and E1124K are not pathogenic in mice. (A) Dorsal surface, coronal forebrain, and sagittal hindbrain views from control and mutant same-sex littermate pairs showed grossly normal brains for R89H homozygous, E1124K homozygous, or R89H/E1124K compound (trans) heterozygous animals on FVB/NJ background. Ratios between same-sex littermates for (B) vermis midline area, (C) average cortical thickness, (D) midline corpus callosum thickness, and (E) weight at sacrifice fail to identify significant deviations for any of these genotypes. Scale bars, 1 mm. (TIF) [file pgen.1009017.s011.tif]

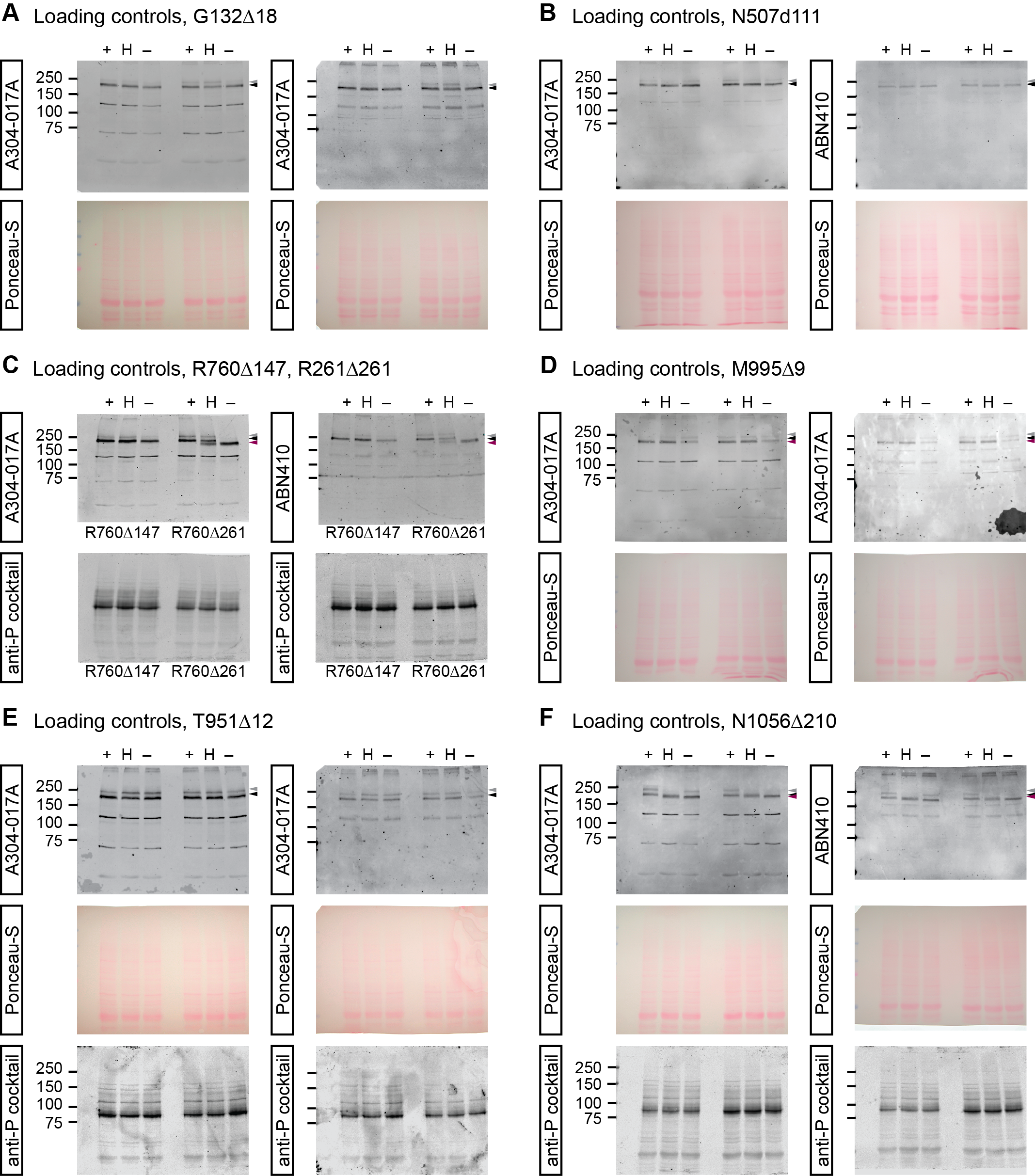

Supplement: S5 Fig — Western blots and loading controls. Full blots and loading control images for in-frame deletion variants G132Δ18 (A), N507Δ111 (B), R760Δ147 and R760Δ261 (C), M995Δ9 (D), T951Δ12 (E), and N1056Δ210 (F). Size marker molecular weight in kDa is shown to the left. Position of the primary Zfp423 band is indicated by a black arrowhead to the right of the blot and the inconsistent conformational isomer by a gray and any consistently observed mutant specific band is indicated by a purple arrowhead. (TIF) [file pgen.1009017.s012.tif]

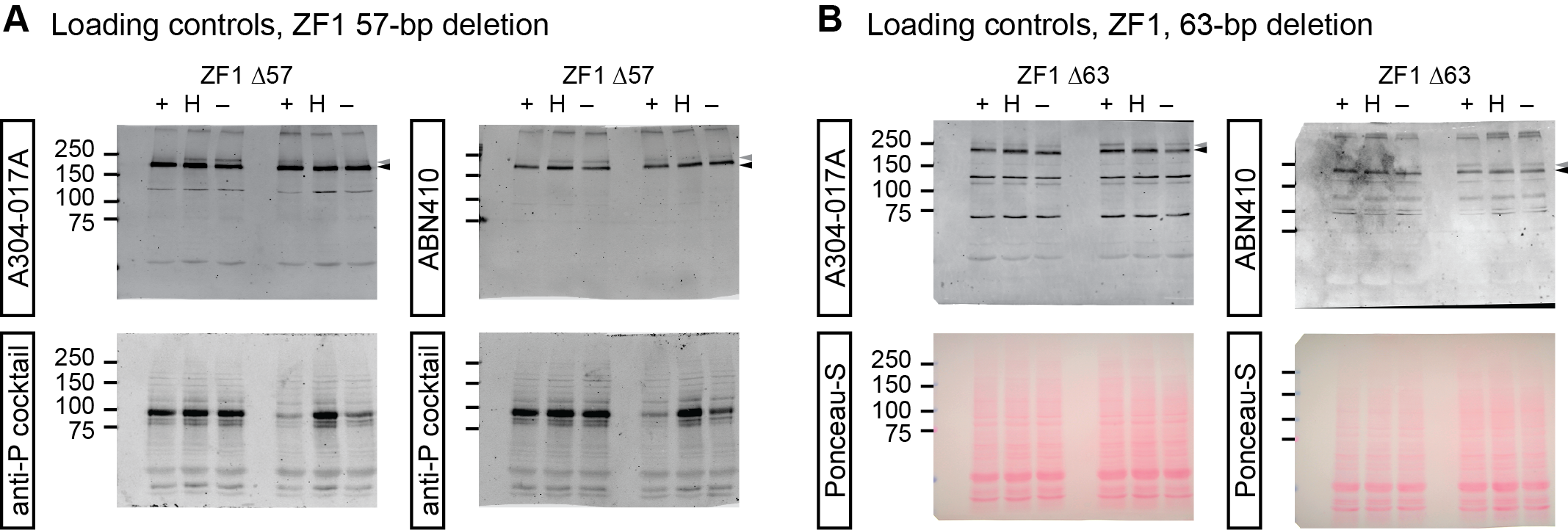

Supplement: S6 Fig — Western blots and loading controls. Full blots and loading control images for 57-bp (A) and 63-bp (B) in-frame deletions of zinc finger 1. Size marker molecular weight in kDa is shown to the left. Position of the primary Zfp423 band is indicated by a black arrowhead to the right of the blot and the inconsistent conformational isomer by a gray and any consistently observed mutant specific band is indicated by a purple arrowhead. (TIF) [file pgen.1009017.s013.tif]
