# Supplementary material for: ZNF423 patient variants, truncations, and in-frame deletions in mice define an allele-dependent range of midline brain abnormalities
Source: PLoS Genet. 2020 Sep 14;16(9):e1009017. doi: 10.1371/journal.pgen.1009017 (PMC7515201; doi:10.1371/journal.pgen.1009017)
Supplement: Attachment — Submitted filename: Response-to-reviewers_Deshpande.pdf [file pgen.1009017.s018.pdf]

Response to Reviewers:
We thank all three reviewers for thoughtful comments and helpful suggestions. Complete reviewer comments are reproduced below with our responses in italics. Reviewer suggestions have strengthened the manuscript, which we believe is now ready for formal publication.

Reviewer #1: The paper submitted by Prof. Hamilton and collaborators describes a comprehensive collection of around 50 mutations in Zfp423, engineered in mice by genome editing, and their phenotypic outcome. Some of the mutations were previously found in patients with neurodevelopmental defects, and some are instead chosen based on their expected effect on the protein function. Mouse has been chosen as an experimental model since ZNF423 and Zfp423 are highly conserved between human and mice (and across all vertebrates). Therefore, this animal model represents a useful, cost-effective tool to validate the effect of gene variants in complex, developing biological systems compared to cell-based models.

The article is interesting both in the specific ZNF423 characterization and, more broadly, in the way it depicts the potential of the engineered animal model tool. Results are overall convincing, and the substantial load of work conducted to gather all the required information is remarkable. However, the language used to throughout the paper is often unclear and suboptimally suited to a scientific context. Major English editing is therefore needed in order to ensure clarity.

We thank the reviewer for appreciating and commenting on the significance of the work. We have edited the revision to improve clarity and usage throughout. All authors, each a native speaker, have reviewed and agreed to the revisions.

Minors:
Abstract: "In-frame deletions of select zinc fingers…" should be "In-frame deletions of selected zinc fingers…"

Select is an adjective as well as a noun. On reflection, however, we have edited this to "specific" as apart from ZF1 the other ZF deletions were incidental to the targeted replacements.

Page 3: "Progress from large reference databases such as ExAC [3], gnomAD [4], and UK Biobank [5] allow powerful statistical evidence against pathogenicity…" should be "Progress from large reference databases such as ExAC [3], gnomAD [4], and UK Biobank [5] allows powerful statistical evidence against pathogenicity…"

Thank you for spotting this. Agreed and changed.

Page 6: "…allowed additional probes of protein stability…" should be rephrased.

Agreed; "additional probes" changed to "observations."

Page 7: "…to detect modest differences in from 10-15 sample pairs…" should be "…to detect modest differences in 10-15 sample pairs…"

Agreed and changed.

Page 9: "To avoid potential confounding of age…" should be "To avoid potential confounding effects of age…"

Agreed and changed.

Page 10: "…in surface photographs (Figure 1D)." Figure 1D doesn't exist; does it refers to figure 1C?

Yes. The figure reference is now corrected.

Page 12: "PTC variants that escape nonsensemediated decay often enough to produce a variant protein can have dominant negative properties by decoupling functional domains [33]." It is quite unclear and should be rephrased

This sentence has been lengthened to "PTC variants whose RNAs escape nonsense-mediated decay often enough to produce detectable levels of variant protein can have dominant negative genetic properties by decoupling functional domains relative to the non-mutant protein" We hope this clarifies the meaning.

Page 12: "In Chaki et al. [7], one of us (B.A.H.) speculated that JBTS19 patients…" can be better rephrased.

It would be simpler to say "Chaki et al. speculated" or "We previously speculated" but those more euphonious constructions obfuscate the blame for this errant speculation, which lies squarely with the senior author of the current ms (B.A.H.). I will own my errors.

Page 15: "The exon 8 deletion produced a nearly full length protein, but one that must lack both histidine…" can be better rephrased

Rephrased as "The exon 8 deletion produced a protein of near-normal size, but one that must lack both histidine…"

Figures:
Fig 1B: blue/black are difficult to distinguish

We have bold-faced the blue hydrophobic residues, shaded the zinc-coordinating residues in grey with white letters, and removed the "-" designation from other residues to simplify add contrast to the figure.

Fig 1C: the figure caption reports "significantly" but no p-value is shown or reported.

Reviewers requested distinct p-values for several panels. We agree that explicit values are helpful guides for strength of evidence, while remembering that p-values are influenced by several factors (empirical distribution, effect magnitude, variance, and sample size) and should be interpreted with caution, especially where multiple hypotheses are considered. We now include a new Supplemental Table S5 with 2018 nominal, FWER-corrected, and FDR-corrected values, listed with sample size and mean ratios, for each phenotype across all tested genotypes.

Fig 2A: all the mutations should be carried in homozygous fashion, but it is better to reiterate this concept in the caption

"Among PTC homozygotes…" added to figure legend.

Fig 4B: the WT seems to have the same isoform carried by the homozygous delta91 (and in higher amount compared to the other); any explanation for that?

The two bands are conformational isomers, not distinct peptide lengths. Wild-type Zfp423 often appears as two protein conformer bands, as we have previously reported in Cho, Hong et al. (2013, PMID 23762491) and Hong & Hamilton (2016, PMID 27727273). Even at high DTT concentrations, the number Cys residues included in 30 C2H2 ZFs and 1 putative C4 ZF, makes denaturation, migration and detection hypersensitive and

somewhat less predictable. The standard 5' boiling protocol for SDS-PAGE, for example, results in complete loss of detection by several anti-Zfp423 antibodies.

Mouse Zfp423 comprises 1292 amino acid positions, of which exon 8 encodes just 9. We would not expect a visible protein size shift due to this mutation and do not see one.

Fig 7D: pink/red and blue are very difficult to spot

We have changed pink to orange for better color separation and boldfaced the residues in blue.

WBs in general: in materials and methods it is explained how quantification and normalization are carried out, but it is critical to have either a quantification graph or a loading control image in each panel (or at least in supplementary figures)

We agree that transparency requires better documentation and thank the reviewer for this suggestion. We now include full images of each blot along with either the Ponceau-S stain prior to antibody or the signal from the anti-phosphoprotein cocktail (or both) as loading controls and a graph of all quantified protein data in new supporting figures S1, S2, S5, and S6. In the course of doing so, we noticed that the image previously shown for H1277Y N-terminal antibody (Figure 5C, lower panel) was from the wrong file and have now corrected this and re-checked and verified each of the other images from this collection. We apologize for the error. In addition, Figure 3C has been edited to correct a typo in the antibody name

Possible in-depht analysis:
in Fig 3A, mutations H88Wfs and H503Qfs seem to have a sex-dependent phenotype (males show mostly and ratio >1, while females show mostly a ratio <1). The same applies to Fig. 3B, for mutations L125Rfs and A1067Vfs (females show mostly a ratio >1, while for males it is always < or around 1). Do the authors have any further evidence of this?

The reviewer has a discerning eye, but these are random fluctuations. That two of 13 heterozygous null genotypes show a sex skew one direction for vermis and two other genotypes show skew in the opposite direction for weight post-hoc without a prior hypothesis or supporting evidence for sex-bias from any of >60 other tested genotypes would be hard to support post-hoc on this evidence.

in Fig 3D, animals with a normalized dosage of around 0,5 showed a worse phenotype compared to those with a normalized dosage of around 1?

This is an interesting idea, but our data do not address it. We expect most of the variation in Western blot from heterozygotes to be measurement error rather than biological variation. As noted above, this is a somewhat challenging protein. We also do not have phenotype data on the neonates from which we made protein extracts. Protein samples were taken from newborn pups prior to genotyping and regrettably were not photographed during dissection.

Did the authors performed any co-IP to prove the retained or lost interaction with known interactors at each mutated site? (e.g., a microdeletion in the SMAD binding domain could or could not affect the SMAD binding and therefore the presence or lack of phenotype, respectively)

We appreciate this suggestion as well. We have planned a more comprehensive approach to this in a pending grant application and hope to extend the work in this direction, but believe it is beyond the scope of the current manuscript.

Reviewer #2: In this report, Deshpande et al generate a substantial number of mouse strains with mutations in the Znf423 locus. These mutations include known or likely pathogenic variants identified in genomic analysis of patients with Joubert Syndrome and related disorders, as well as variants of uncertain significance. Additional mutant strains were analyzed with various premature termination/frameshift mutations and in-frame deletions. The team leverage the capacity of CRISPR-mediated mutagenesis to generate these models.

The overall impetus of this study is multifold. The efforts leverage existing knowledge of the null phenotypes from previous Znf423 knockout studies to identify somewhat superficial but sensitive and rapidly scorable phenotypes (eg size of the vermis) as a means of determining the phenotypic severity of a given mutation. This is a logical means for quickly assessing phenotypes, which is necessary for the scale of the number mutant lines being studied. From this work the team identify multiple patient-derived variants as being deleterious in the animal model, and other variants as non-phenotypic. Alleles that generate domain-specific deletions also highlight potential ZNF423 protein interaction partners (eg SMADs) and the context-dependency on these interctions. The use of western blotting to assess protein stability is another important feature of the report that provides important information on the nature of different variants. The report will logically lead to further studies in the phenotypes of many of the models generated, which will be an important further studies for Joubert Syndrome.

This substantial effort also highlights the capacity for rapid (relatively) generation of these mouse models and the capacity for relevant high level phenotypic analysis to assess the pathogenicity of many clinically identified variants. This is an important advancement in studies of novel and rare genetic variants identified in human genomics efforts for pathogenicity and causation. There has been concern in some areas of the functional genomics field in the use of the mouse for assessment of variant function; this work is an excellent case study in the scalable capacity for using the mouse in this context. This study will be both of interest to those studying ZNF423 and Joubert Syndrome and also for geneticists with interest in advancing functional genomics studies.

We thank the reviewer for this thoughtful summary and supportive comments on the potential impact of the work.

Overall the manuscript is complete and well-written, and the conclusions are consistent with the data presented. Some minor clarifications would be beneficial for the broader readership. Some suggested minor edits:

1. Figures in general.
a. The figures are dense and packed with data. Some smaller panels suffer from a lack of clarity due to their small size. Specific issues for each figure are noted below. The authors may want to consider summarizing the quantitative data and presenting only some representative images, rather than presenting a panel for each mutant cerebellum, for example. Some of the more extensive panels can be shifted to Supplemental data.

We thank the reviewer for this thoughtful comment. We have responded to specific issues where noted below. On the general point, we expect that full rendering in page layout and the zoom tool in PLOS online figure format may resolve some of the density relative to fixed page width in the pdf file. We have purposefully tried to minimize supplemental assignments, however, where the figure is needed to understand the work.

Supplements must be accessed separately from the main paper, which puts an additional barrier between the typical reader and the data. We will do what is needed to publish, but prefer to keep the figure components together reserve supplements for primary data that allow reuse or reanalysis by others for figures that illustrate further examples of data already given in the main figures.

b. An image or diagram indicating where the vermis measurement is done would be helpful

We thank the reviewer for this helpful comment. This has now been marked on the control littermate image in Figure 2 and commented in the Figure 2 legend.

2. Supplemental data in general. The Supplemental data contains a substantial amount of fairly raw data. Although useful, some of the data is somewhat too raw and contains lab-specific jargon that makes it difficult to understand. For example, in Table S1, the Results column contains a lot of raw notes on the injection outcome that should be clarified and made more consistent. It is not clear what the difference between 'edited' and 'mutated' is, and this should be clarified. 'NTBR' is not defined. These should be cleaned up and made more clear.

We have appended Supporting Table Captions that better describe the contents of each table. Our goal is to make the primary data available for scrutiny, re-analysis, or re-use by other scientists. NTBR was a vestigial notation that the injection Needs To Be Repeated; as this was redundant with comments that point to date repeated, all instances of NTBR in Table S1 have been removed. The single instance of "mutated" has been revised to "edited" as these were used interchangeably.

3. Fig 1C – Please clarify that most of the graph consists of analyses of heterozygotes

Except for the first two samples (non-mutant control and PTC heterozygotes), all data in Figure 1C are comparisons between homozygotes for the named allele and their wild-type same-sex littermates. This is now explicit in the figure legend.

4. Fig 2B – report individual Chi squares for each genotype offspring data for each mutation

*We disagree. Owing to the retrospective nature of sampling for this measure (which can inflate variation between lines due to time-dependent sampling in the work flow) and unequal sample sizes (hence unequal power), prominently displaying the p-value in the figure would give a false impression of variation among the different PTC variants (and in this context false negatives are both a larger problem than false positives and one much harder to correct for in an easily read format). We strongly prefer to display actual counts. This allows anyone who wishes to perform another analysis. The segmented bar plots better represent the direction and magnitude of skew than the Chi-square values across functionally equivalent alleles and we believe that the aggregate p-value is therefore the more important statistic to emphasize for this figure. We have, however, fixed a typographical error in the N1056Qfs29 and A1067Vfs*51 mutant counts (values had been inadvertently switched between the two).*

5. Fig 2D clarify in the figure legend that these ratios measures are of homozygous mice.

We have clarified this in the text paragraph that references the figure, as it applies to all panels of Figure 2.

6. Fig. 3A,B. It would be helpful if individual significance calculations (p value) for each mutant, perhaps in a supplemental table.

As noted in response to Reviewer 1, we now include Supplemental Table S5 with 2018 nominal, FWER-corrected, and FDR-corrected values listed with sample size and mean ratios for each phenotype across all tested genotypes 7.

Fig 3. The reported increased in protein level in hets suggests either negative regulatory loop, or alteration of cell type proportions in het mice. Although a minor point, additional supporting experiments would be helpful. For example, RNA from brains could be examined for transcript steady state levels of RNA from wt allele.

Our best estimate of the Zfp423 protein level in heterozygous animals is a bit more than 50% of control littermate (0.56-0.73 with the more reliable A304-017A antibody, Figure 3D). We agree that additional experiments here, particularly spatial transcriptomics that could disentangle cellular RNA level from cell composition, would be informative. However, we are not able to do this at present and with laboratory access limited to ≥25% pre-pandemic levels we cannot estimate when this new line of study would be achievable. As the present data already represent several years of concerted effort, we believe that the delay to add this experiment would not be justified.

8. Fig 4B. the western is not particularly convincing, partly due to the small size of the panel. Additionally, multiple non-specific bands are apparent, and it appears that multiple bands are affected in the homozygous mutants, including a band the authors do not indicate is ZFP423. The presence of a lane which includes lysate from a bonafide null brain, such as a lysate from one of the several frameshift or nonsense mice used in Fig 2A, would allow the reader to more clearly identify which bands are non-specific, and which are indicative of ZFP423.

Figure 4B includes duplicate blots with two distinct antibodies against Zfp423 and that both show reduced amount of ~normal length protein, consistent with loss of only 9 amino acids in exon 8. Each antibody does recognize additional bands that are irrelevant to Zfp423, as documented in Figure 2A for 11 distinct frameshift mutations at spread across the open reading frame from codon 125 to codon 1056 out of 1284. The N-terminal A304 antibody had both higher signal and lower background than ABN410 at usable titers in our hands, but both contribute to the evidence. In all panels, bona fide and reproducible Zfp423 bands are indicated by the registration lines and labels at the side of the image.

9. Fig. 6D. Individual statistical measures of significance should be reported. Given that the authors suggest some alleles are more severe and correlate with protein levels, these individual measures should be indicated.

As noted in response to Reviewer 1, we now include a new Supplemental Table S5 with 2018 nominal, FWER-corrected, and FDR-corrected values listed with sample size and mean ratios for each phenotype across all tested genotypes. Protein quantification from blots is an art that relies on several assumptions; we have nonetheless made good-faith estimates from infrared image analyses and graph these data in new supplemental figure S1_Fig, panel C.

10. Materials and Methods. Clarify how the brain tissues were fixed, when used, for the gross brain measures. The figure legend of Fig 1 indicates both fresh and fixed tissues were analyzed.

We have added this information to the methods section under the "Anatomical measures" heading.

Reviewer #3: In this paper, the authors tackle a key point in personal genomics, namely the reliability of in silico predictions regarding a mutation's clinical impact based on criteria including evolutionary constraints or the physic-chemical consequences of aminoacid substitutions.

They apply their validation strategy to the study of ZNF423/Zfp423, a gene of high relevance in cerebellar development that has been implicated in rare cases of Joubert syndrome, cerebellar vermis hypoplasia and extraneural disorders, including nephronophthisis. The gene also plays key roles in adipocyte development and encodes a protein that acts both as a transcription factor and as a hub for the assembly of multiprotein complexes implicated in key developmental pathways. The protein plays important roles in the regulation of ciliary functions and DNA damage repair.

Contrary to the popular trend of analyzing the effects of mutations in human IPSC-derived neurons, often of an incompletely defined nature, isolated from their extracellular matrix environment, cell-cell interactions and circuit connections, and prone to irreproducible behaviors, they take a systematic in-vivo approach creating a large allele series in the mouse by CRISPR-Cas9-based genome editing. To gauge the effects of these mutations, they resort to simple and significant parameters, which include the size of the cerebellar vermis and of forebrain commissural tracts. In addition, they use motor coordination tests to charaterize mouse behavior.

This paper sets an innovative standard for the systematic functional analysis of gene variants. I expect that it will be widely cited and that it may constitute a cornerstone in the field of functional genetics.

The experiments described in this paper are sound, well presented and appropriately controlled. The results are of very high quality. The paper is well written and the results are thoroughly, yet concisely discussed.

I have no major criticism to express. As a minor point, I suggest that the authors spell the mouse protein in all capitals, which is more appropriate for a genetics journal. Fortunately, in this case, human and mouse homologs have different acronyms.

We thank Reviewer 3 for articulating support for this work. While we prefer to keep gene and protein names distinct by italics rather than all capitals, as we have for earlier papers on Zfp423, we agree to follow house style from the journal.
